# Depression among Elderly of Chhayanath Rara Municipality, Mugu, Nepal: An Observational Study

**DOI:** 10.31729/jnma.8885

**Published:** 2025-02-28

**Authors:** Nabina Malla, Lisasha Poudel, Sushma Pokhrel, Vidya Chaudhary, Prajita Mali

**Affiliations:** 1 Institute for Implementation Science and Health, Naya Thimi, Bhaktapur, Nepal; 2 Mero job limited, Kathmandu, Bagmati Province, Nepal; 3 Lambton College, Toronto, Canada; 4 Kathmandu University School of Medical Sciences, Dhulikhel, Kavre, Nepal

**Keywords:** *depression*, *elderly*, *prevalence*

## Abstract

**Introduction:**

Depression is an important public health issue responsible for considerable morbidity and mortality among elderly. There were very few studies related with depression among elderly from rural community of Nepal. The objectives were to assess depression among elderly in Chhayanath Rara municipality.

**Methods:**

An observational cross-section study was conducted among 387 elderly through face-to-face interviews by using Geriatric Depression Scale-short scale (GSD 15) in a municipality of Nepal. Ethical Approval was taken from Institutional Review Committee (Reference Number: 079/80-015 ). Data were entered and analyzed into SPSS version 20. Descriptive statistics was employed to assess the prevalence of elderly depression.

**Results:**

The prevalence of depression among elderly was found to be 282 (72.87%; 95% CI: 68.44%-77.30%). Of those, 124 (32.04%) experienced mild depression, 115 (29.72%) had moderate depression, and 43 (11.11%) suffered from severe depression.

**Conclusions:**

The prevalence of depression in elderly was found higher compared to previous studies and other population.

## INTRODUCTION

The older population have been significantly increased in both developed and developing countries since the 20th century.^1^ The World Health Organization (WHO) has estimated that between 2015 and 2050, the proportion of the elderly population will almost double, which is 12% to 22%.^2^ Depression is the most common problem in the elderly population and is a major public health problem.^3^ Its prevalence ranges between 10% and 20%, depending on cultural situations.^4^ Depression is problematic in developing countries because there is a lack of exact prevalence, scope of disease and resource to address such problems.^5^

The life expectancy in Nepal has increased from 62.39 in 2000 to 70.25 in 2016. Due to aging dynamics, there are around 2.1million elderly inhabitants, which consist 8.1% of total population of Nepal Increasing in elderly population carries a disease burden and country possess serious challenges to available health services.^6,7^

Depression in elderly is one of the important health issues with the prevalence of 25.5% to 60.6% in community, however the status of depression among elderly in rural community is unknown.^8^ so, this study aimed to assess the depression among elderly of Chhayanath Rara Municipality, Mugu, one of Nepal's rural communities.

## METHODS

An observational cross-section study was carried out to assess the depression among elderly of Chhayanath Rara Municipality, Mugu, Nepal. The district has total population of 55,286, of which 3,936 were elderly population.^9^ Mugu, located in Karnali Province, is among the districts with low Human Development Index (HDI) 0.397.^10^ Furthermore, its mountainous terrain poses challenges such as limited access to the basic health services and frequent exposure to environmental hazards, which significantly impact population health, particularly among the elderly. The study duration was from March to December 2019. Data was collected between August to September 2019.

The ethical clearance was taken from the Nepal Health Research Council, Ethical Review Board (Reference number: 371/2019).

Senior Citizen was defined as citizen who are completed the age of 60 and above based on Nepal senior citizen Act, 2063.^11^ The elderly who were residing in Chhayanath Rara Municipality providing consent were included in the study. However, elderly individuals diagnosed with mental illness such as dementia, psychosis, or depression (confirmed through a review of their prescriptions), as well as those with physical conditions such as hearing impairment, and dumbness, were excluded. The sample size was calculated using following formula:


$$\begin{array}{l} \begin{array}{ll} \text{n} & ={\text{Z}}^{2}\times \frac{\text{p}\times \text{q}}{{\text{e}}^{\text{2}}}\\ & =1.96^{2}\times \frac{0.355\times (1-0.355)}{0.05^{2}} \end{array}\\ \text{n }=352 \end{array}$$

Where:

Z represents the z-value (1.96) at a 95% Confidence Interval (CI).
P is the estimated prevalence of depression in elderly (35.5%).^12^
q = 1-p
e is the margin of error (5%).
Now, considering a 10% of non-response rate, sample size of this study was determined to be 387.

The sample of 387 was selected by using convenient sampling technique. However, only one elderly individual was chosen from each household. Verbal and written consent was taken from participants. The participants were interviewed face to face by the researcher.

The tool used for data collection in the study was Semi-structured questionnaire adopted from Geriatric depression scale (GDS), Instrumental activities of daily living (IADL tool) and Nepal Demographic and Health Survey (NDHS) Wealth Index.^13,14^

The GDS short form consisted of the 15-item questionnaire in which participant was asked to respond by answering yes or, no about how they felt over the past week. For interpretation of depression, score (0 - 4 as normal), (5 - 8 as mild depression), (9 - 12 as moderate depression), (13 - 15 as severe depression. The questionnaire was classified according to various categories based on literatures and standard levels; it was divided into socio-demographic characteristics, socio-economic status of household, predisposing factors and GDS scale.^13^

The questionnaire was translated into the Nepali language. The pre-test of the study was done by taking 10% of the sample size among elderly of Kubende, Sindupalchok. Cronbach alpha was calculated of the translated questionnaire, which was 0.78.

Data were entered and analyzed in Statistical Package for the Social Sciences version 20. Descriptive statistics was used to assess the prevalence of elderly depression.

## RESULTS

Among all the participants, prevalence of depression was found to be 282 (72.87%; 95% CI: 68.44-77.30), (Table 1).

**Table 1:** Distribution of respondents according to prevalence of depression and its level (n=387).

Patient demographics	Mean± SD/Number (Percentage)
**Prevalence of Depression**	
Normal	105 (27.13)
Depression	282 (72.87)
**Level of Depression**	
Mild	124 (32.04)
Moderate	115 (29.72)
Severe	43 (11.11)

**Table 2:** Socio-demographic distribution of the participants (n=387).

Characteristics	n (%)
**Age in years**	
60-70	286 (73.90)
71-80	83 (21.45)
81-90	18 (4.65)
**Gender**	
Male	182 (47.03)
Female	205 (52.97)
**Ethnicity**	
Dalit	102 (26.36)
Brahmin/Chhetri	245 (63.31)
Others	40 (10.33)
**Marital status**	
Married	198 (51.16)
Unmarried	3 (0.78)
Widow	184 (47.55)
Separated	2 (0.52)
**Family type**	
Nuclear	73 (18.86)
Joint	314 (81.14)
**Educational status**	
Illiterate	335 (86.56)
Literate	52 (13.44)
**Living arrangement**	
Alone	33 (8.53)
With spouse only	40 (10.34)
With children only	155 (40.05)
Both spouse and children	157 (40.57)
Others	2 (0.52)
**Source of income**	
Agriculture	68 (17.57)
Business	7 (1.81)
Daily wages	4 (1.03)
Pension	10 (2.58)
Old age allowance	54 (13.95)
Dependent in family	244 (63.05)

**Table 3:** Socio-economic distribution of the participants (n=387).

Wealth quintile	n (%)
Lowest	77 (19.88)
Second	82 (21.20)
Middle	65 (16.79)
Fourth	86 (22.22)
Highest	77 (19.89)

**Table 4:** Predisposing Factors among participants (n=387).

Variable	n (%)
**Role within family**	
Decision maker	10 (2.58)
Views get importance	295 (76.22)
Neglected	82 (21.20)
**Sleep disturbance**	
Yes	211 (54.52)
No	176 (45.48)
**Loneliness**	
Often	17 (4.39)
Sometime	207 (53.49)
Rarely	46 (11.88)
Never	117 (30.24)
**Adequate rest**	
Yes	207 (53.48)
No	180 (46.52)
**IADL**	
Dependent	55 (14.21)
Independent	332 (85.78)

IADL: Instrumental activities of daily living

Amongst the total participants 286 (73.90%) were in age group 60-70 years, 205 (52.97%)%) were male and 245 (63.31%) were Brahmin/Chettri ethnic group. Similarly, the population studied consisted of 198 (51.16%) married, 314 (81.14%) nucler family, 335 (86.56%) illiterate, 157 (40.57%) living with both spouse and children and 244 (63.05%) dependent in family (Table 2).

In socio-economic distribution, the wealth quintile was lowest in 77 (19.88%), (Table 3).

Among 387 elderly individuals, 295 (76.22%) reported their views were given importance within the family, while 82 (21.20%) felt neglected. Sleep disturbances were reported by 211 (54.52%), and 207 (53.49%) experienced loneliness sometimes. Additionally, 207 (53.48%) reported having adequate rest, and 332 (85.78%) were independent in instrumental activities of daily living (IADL) (Table 4).

## DISCUSSION

The findings of thisstudy highlight a significantprevalence of depression 282 (72.87%) among theelderly population in Chhayanath Rara Municipality, as measured by the Geriatric Depression Scale (GDS). Systematic reviews have shown that depression rates are generally higher in rural areas compared to urban settings. However, the observed prevalence exceeds the range of 25.5% to 60.6% reported in systematic reviews of community-based studies conducted in Nepal.^8^ Similarly, a study from India reported a broader range of 6% to 53.7%, while a study in Korea exhibited a prevalence of 63% among the elderly.^5,15^ These disparities may result from differences in study design, sampling methods, cultural norms, and criteria used to define and measure depression.

Our study reported a high illiteracy rate of 335 (86.56%) among the elderly population we included in the study, whereas a study conducted in Nepal exhibited a lower illiteracy rate of 22.4%.^5^ Various studies suggest that the education level of participants may determine the prevalence of depression. This might be due to the fact that most literate individuals are financially independent, participate in decision-making processes, and are more aware of their health, leading to better health-seeking behavior compared to illiterate individuals.

Living arrangements and roles within the family also determine the presence of depression. Our study showed that 157 (40.57%) of participants lived with both their spouse and children, while only 10 (2.58%) acted as decision-makers within their family. However, a study done in Kathmandu revealed that almost all (95.7%) lived with family or a spouse, and 74.1% acted as the head of the family.^16^ These differences might be due to variations in cultural, economic, and demographic factors. In Kathmandu, strong cultural norms may emphasize extended family living and respect for elders, contributing to leadership roles for the elderly. Additionally, the availability of job opportunities in urban areas like Kathmandu may enhance family stability and intergenerational cohabitation.

The feeling of loneliness is another important factor for depression in this study, with 224 (57.88%) of participants often or sometimes feeling lonely. A study done in Koshi Zone reported that only 18% of participants felt lonely.^17^ This might be due to the fact that our study had 184 (47.55%) widowed participants, whereas the study in Koshi Zone had 22%. The higher depression among widows/widowers may be attributed to the lack of a partner to share emotional and social support.

Having functional disabilities in instrumental activities of daily living (IADL) is also a factor for depression in many studies. Findings from other studies^5,15^ were consistent, which contradicts this study. In our study, 332 (85.78%) of participants were independent in performing IADL, reflecting a degree of functional ability despite psychological challenges. This might be because they were obliged to engage in household activities rather than being dependent on their children.

A previous study conducted in Eastern India showed a strong relationship between depression and socioeconomic status, noting that individuals from higher wealth quintiles could fulfill their requirements and seek health services independently compared to those with lower socioeconomic status.^18^ Similarly, our study found that 224 (57.87%) of households fell under the middle, second, and lowest wealth quintiles, which may explain the higher prevalence of depression observed.

The study has some limitations. Firstly, a convenient sampling technique was used to select the study population. Additionally, the study was cross-sectional and conducted in one municipality, so the findings cannot be generalized to other areas of Nepal.

## CONCLUSIONS

The study reveals a high prevalence of depression among the elderly population, with a significant proportion experiencing symptoms ranging from mild to severe. These findings highlight the need for greater attention to mental health issues in this age group, particularly in rural settings where resources may be limited. While the study does not propose specific interventions, it emphasizes the importance of understanding and addressing depression as a critical health concern among the elderly.
